# Analysis of chi angle distributions in free amino acids via multiplet fitting of proton scalar couplings

**DOI:** 10.5194/mr-5-103-2024

**Published:** 2024-08-19

**Authors:** Nabiha R. Syed, Nafisa B. Masud, Colin A. Smith

**Affiliations:** Department of Chemistry, Wesleyan University, Middletown, CT, United States

## Abstract

Scalar couplings are a fundamental aspect of nuclear magnetic resonance (NMR) experiments and provide rich information about electron-mediated interactions between nuclei. ${}^{3}J$ couplings are particularly useful for determining molecular structure through the Karplus relationship, a mathematical formula used for calculating ${}^{3}J$ coupling constants from dihedral angles. In small molecules, scalar couplings are often determined through analysis of one-dimensional proton spectra. Larger proteins have typically required specialized multidimensional pulse programs designed to overcome spectral crowding and multiplet complexity. Here, we present a generalized framework for fitting scalar couplings with arbitrarily complex multiplet patterns using a weak-coupling model. The method is implemented in FitNMR and applicable to one-dimensional, two-dimensional, and three-dimensional NMR spectra. To gain insight into the proton–proton coupling patterns present in protein side chains, we analyze a set of free amino acid one-dimensional spectra. We show that the weak-coupling assumption is largely sufficient for fitting the majority of resonances, although there are notable exceptions. To enable structural interpretation of all couplings, we extend generalized and self-consistent Karplus equation parameterizations to all χ angles. An enhanced model of side-chain motion incorporating rotamer statistics from the Protein Data Bank (PDB) is developed. Even without stereospecific assignments of the beta hydrogens, we find that two couplings are sufficient to exclude a single-rotamer model for all amino acids except proline. While most free amino acids show rotameric populations consistent with crystal structure statistics, beta-branched valine and isoleucine deviate substantially.

## 1 Introduction

The structure and dynamics of amino acid side chains are often critical for protein function. Side chains are not only an important part of the folded structure of proteins, but also key in facilitating molecular recognition, allosteric regulation, and catalysis. Nuclear magnetic resonance (NMR) is a particularly powerful technique for studying side chains as they move in solution at physiological temperatures. ${}^{3}J$ scalar couplings give the most direct information about the local structure of side chains through the mathematical relationship between the dihedral angles of rotatable bonds and ${}^{3}J$ , which was originally formulated by [Bibr bib1.bibx34]. The numerous NMR experiments for measuring protein scalar couplings have been reviewed in detail by [Bibr bib1.bibx58]. Notably, it is possible to measure every scalar coupling involved in the side-chain $\chi_{1}$ angle, including ${}^{3}J$ (HA–HB), ${}^{3}J$ (C–HB), ${}^{3}J$ (N–HB), ${}^{3}J$ (HA–CG), ${}^{3}J$ (N–CG), and ${}^{3}J$ (C–CG). (Protein Data Bank, PDB, atom names are used throughout this paper.)

Homonuclear proton–proton couplings, which are the focus of the present study, result in sometimes complex multiplet patterns in one-dimensional proton NMR spectra. Numerous pulse sequences have been developed to overcome this complexity and make proton–proton couplings easier to resolve and quantify in multidimensional spectra. The first was Exclusive Correlation Spectroscopy (E.COSY) [Bibr bib1.bibx20], which generates cross-peak multiplets with reduced numbers of peaks and takes advantage of passive couplings to make line splitting by the active coupling visible for inspection and quantification. For ${}^{13}C$ -labeled samples, this idea was extended using modified versions of the HCCH-COSY and HCCH-TOCSY experiments, where ${}^{1}J$ (C–H) was used to resolve ${}^{3}J$ (H–H) [Bibr bib1.bibx18]. An HXYH experiment further improved experimental efficiency by simultaneously measuring both backbone and side-chain ${}^{3}J$ couplings using ${}^{13}C^{15}N$ -labeled proteins [Bibr bib1.bibx53]. Another class of experiments, quantitative J correlation, uses the ratio between a diagonal and cross-peak to determine the value of the coupling constant, first demonstrated with the HNHA experiment, which measures the backbone ${}^{3}J$ (H–HA) [Bibr bib1.bibx57]. A HACACB-COSY adaptation of this technique enabled quantification of side-chain couplings [Bibr bib1.bibx23].

Another approach for obtaining scalar couplings uses numerical processing of a pair of matched experimental spectra, one having in-phase peaks with the same sign and the other having anti-phase peaks with opposite signs [Bibr bib1.bibx42]. In these methods, a pair of trial anti-phase and in-phase peaks are convolved with a multiplet in the respective spectra. The coupling is determined by finding the separation between the trial peaks that results in maximum agreement between the two convolved spectra. However, peak overlap can be a problem with these types of methods because only a single multiplet is analyzed at a time.

Various approaches have been applied to directly fit peak multiplets that can handle peak overlap. SpinEvolution [Bibr bib1.bibx56], Quantum Mechanical Total Line Shape (QMTLS) fitting in PERCH (PERCH Solutions), ChemAdder [Bibr bib1.bibx54], Guided Ideographic Spin System Model Optimization (GISSMO) [Bibr bib1.bibx10], ANATOLIA [Bibr bib1.bibx9], and Cosmic Truth (NMR Solutions) [Bibr bib1.bibx1] enable the fitting of a one-dimensional spectrum by iteratively optimizing parameters used to simulate the spectrum by a quantum mechanical description of the spin system(s) [Bibr bib1.bibx6]. Such calculations account for cases where the chemical shift difference between two nuclei approaches the value of their scalar coupling. This leads to strong coupling and the so-called “roofing effect”, where peaks in the multiplet closest to the other nucleus increase in intensity and those farthest decrease in intensity. While such calculations are usually computationally intensive, methods have been developed to very rapidly simulate one-dimensional spectra [Bibr bib1.bibx7]. Global Spectral Deconvolution (GSD) in Mnova NMR (Mestrelab Research) enables fitting individual peaks in one-dimensional spectra and classification of peaks into multiplets [Bibr bib1.bibx3]. More recently, deep neural networks have been combined with line shape fitting to automatically quantify peaks in one-dimensional spectra [Bibr bib1.bibx37].

Several methods exist for fitting multidimensional spectra, including PINT [Bibr bib1.bibx2] and INFOS [Bibr bib1.bibx48], but those tools do not explicitly model scalar couplings. Amplitude-Constrained Multiplet Evaluation (ACME) was developed to fit proton–proton scalar couplings in COSY cross-peaks [Bibr bib1.bibx13]. Explicit modeling of scalar couplings in multidimensional spectra can also be done in Spinach [Bibr bib1.bibx30], which is a widely used software library optimized for simulations of large spin systems. However, like the commercially available NMRSim (Bruker) that can also simulate multidimensional spectra, the calculations can be time-consuming and are not typically used for direct spectral fitting.

Once accurate ${}^{3}J$ couplings have been measured and quantified, they can be interpreted using the Karplus relationship, which relates ${}^{3}J$ to a linear combination of cos⁡θ and either cos⁡2θ or ${\mathrm{cos}}^{2}\theta$ , where θ is the dihedral angle between the coupled nuclei. The three coefficients (a constant and two scaling factors for the cos⁡ functions) determine the Karplus parameterization. The coefficients for proteins have been most often determined using a large set of scalar coupling measurements for which coordinates from X-ray crystallography are also available. A structure-free approach to parameterizing scalar couplings was developed by [Bibr bib1.bibx46]. It depends on the measurement of many different scalar couplings, each with a different relationship to the overall dihedral angle. By having many scalar couplings, both the dihedral angles of the chemical bonds and the associated Karplus parameters can be determined in a self-consistent manner. This approach was originally applied to scalar couplings in the protein backbone [Bibr bib1.bibx46] and then expanded to side chains [Bibr bib1.bibx44]. A model known as the generalized Karplus equation was parameterized 2 decades earlier primarily using data from small molecules with six-membered rings [Bibr bib1.bibx24]. This approach used a formula incorporating differences in the electronegativity between hydrogen and the substituted heavy atoms, the orientation of the substituent relative to the hydrogen, and the electronegativities of secondary substituents.

One of the first studies into the conformational preferences of amino acids using scalar couplings examined the effect of N- and C-terminal charge states on the rotamer equilibrium [Bibr bib1.bibx43]. For individual amino acids, the ContinUous ProbabIlity Distribution (CUPID) method was developed that also incorporated information from nuclear Overhauser effect experiments [Bibr bib1.bibx16]. That and numerous other methods for analyzing scalar coupling data to determine dihedral angles and distributions have been reviewed [Bibr bib1.bibx36]. Scalar couplings are also used to determine conformational ensembles of proteins using the standard and generalized Karplus equations [Bibr bib1.bibx51]. In the context of full proteins, molecular-dynamics-enhanced sampling techniques have been shown to improve convergence and fit to experimental data [Bibr bib1.bibx50]. Beyond scalar couplings, residual dipolar couplings have also been used to analyze side-chain conformations in folded proteins [Bibr bib1.bibx40].

The ${}^{3}J$ (H–HA) scalar coupling is dependent on the phi backbone dihedral angle and takes distinct values depending on whether the residue is part of an alpha helix or beta sheet. In most heteronuclear NMR spectra, the power output required for decoupling ${}^{13}C$ and/or ${}^{15}N$ in isotopically labeled proteins limits the direct-dimension acquisition time, leading to signal truncation that hinders the resolution of the ${}^{3}J$ (H-HA) line splitting. Increasing molecular size also broadens the line widths, further exacerbating the resolution. However, we recently showed that through very precise modeling of signal truncation and apodization, ${}^{3}J$ (H-HA) could be quantified in the ordinary ${}^{1}H{-}^{15}N$ two-dimensional spectra using nonlinear least-squares fitting in FitNMR [Bibr bib1.bibx14]. A byproduct of this fitting is that the ${}^{1}H$ transverse relaxation rate, $R_{2}^{*}$ , can also be quantified, which can provide valuable information about protein structure and dynamics [Bibr bib1.bibx15].

Towards the ultimate goal of being able to similarly quantify side-chain ${}^{1}H$ scalar couplings and $R_{2}^{*}$ values directly from multidimensional spectra of folded proteins as well as extract accurate volumes for clearly overlapping peaks, we present an analysis of the proton–proton couplings in ${}^{1}H$ spectra of individual amino acids. We describe how FitNMR was enhanced to directly model complex multiplet patterns in multidimensional spectra using a simple tabular input/output format. The strengths and weaknesses of using a model that assumes purely weak-coupling interactions are illustrated. To obtain Karplus parameters, we extend a self-consistent parameterization of ${}^{3}J$ (HA–HB) couplings to include ${}^{3}J$ (HB–HG), ${}^{3}J$ (HG–HD), and ${}^{3}J$ (HD–HE). Finally, we apply an enhanced model of side-chain motion incorporating prior rotameric information to determine differences in the conformational preferences between the side chains of free amino acids and those found in crystal structures.

## 2 Methods

### 2.1 Fitting couplings in multidimensional spectra

FitNMR [Bibr bib1.bibx14] was originally designed such that each peak in a multiplet would be a distinct entity. To allow for scalar couplings, the chemical shifts in a given peak could be made a linear combination of auxiliary chemical shift parameters, whose coefficients were chosen such that a scalar coupling in hertz could be mapped onto the parts per million scale. While this functioned well for fitting simple doublets found in protein ${}^{1}H{-}^{15}N$ two-dimensional spectra, it did not scale well to other applications, especially complicated spectra with heterogeneous coupling patterns.

**Table 1 Ch1.T1:** Isoleucine resonance table.

	x	x_sc	1_m0
HA	HA	HA–HB	859348095
HB	HB	HA–HB HB–HG12 HB–HG13 HB–HG2 HB–HG2 HB–HG2	978275274
HG12	HG12	HB–HG12 HG12–HG13 HG12–HD1 HG12–HD1 HG12–HD1	1099447740
HG13	HG13	HB–HG13 HG12–HG13 HG13–HD1 HG13–HD1 HG13–HD1	1088697294
HG2	HG2	HB–HG2	3413052104
HD1	HD1	HG12–HD1 HG13–HD1	3213059843

To address this, we developed a new way of defining NMR spectral features, for which we use the term resonances. They are defined in a comma separated values (CSV) resonances text file, with an example for isoleucine shown in Table [Table Ch1.T1]. The first column gives the name of the resonance, which can be arbitrarily defined. FitNMR supports up to four spectral dimensions, referred to using the names x, y, z, and a, following nomenclature used by NMRPipe [Bibr bib1.bibx12]. The particular nucleus associated with each dimension is given in the column with the same name as the dimension. Scalar couplings active in each dimension are given in a corresponding column whose name has the _sc suffix. They are space-delimited and can also be arbitrarily named, although no nucleus and scalar coupling may share the same name. A scalar coupling can appear several times to produce canonical multiplets like triplets and quartets. For instance, in isoleucine, the HB resonance definition produces a doublet of doublets of doublets of quartets, with couplings to HA, HG12, and HG13 each producing a doublet and couplings to the HG2 methyl group producing a quartet. Additional columns give the volumes associated with individual spectra, referred to by FitNMR as m0 (initial magnetization).

**Table 2 Ch1.T2:** Isoleucine nuclei table.

	omega0_ppm	r2_hz
HA	3.595	0.565
HB	1.908	0.772
HG12	1.396	0.715
HG13	1.187	0.696
HG2	0.936	0.654
HD1	0.864	0.637

**Table 3 Ch1.T3:** Isoleucine scalar couplings table.

	hz
HA–HB	3.95
HB–HG12	4.83
HB–HG13	9.29
HB–HG2	7.02
HG12–HG13	-13.49
HG12–HD1	7.47
HG13–HD1	7.36

Each nucleus referred to in the resonances table is defined in the nuclei table, with an example for isoleucine shown in Table [Table Ch1.T2]. The first column gives the nucleus name. The second, omega0_ppm, column gives the chemical shift offset, $\Omega_{0}$ , in parts per million. The third r2_hz column gives the transverse relaxation rate (including an inhomogeneous contribution), $R_{2}^{*}$ , in hertz. The coupling table (Table [Table Ch1.T3]) just has a single hz data column, with the value of the scalar coupling in hertz. Because they are associated with saturated carbons, all ${}^{2}J$ couplings are assumed to be negative. However, in the present work, the sign has no impact because all couplings are in phase. CSV files for all fitted parameters are available in the data/fit1d_fitnmr_output.tar.gz file within the Supplement ZIP archive.

### 2.2 Fitting amino acid one-dimensional spectra

Starting parameters for fitting amino acid one-dimensional NMR spectra were adapted from the Guided Ideographic Spin System Model Optimization (GISSMO) database [Bibr bib1.bibx10], with couplings added or removed as appropriate. Chemical shifts were manually altered to account for differences in referencing and effects of strong coupling, which FitNMR does not currently model. Standard PDB atom names were used. When two nonmethyl protons were modeled with a single chemical shift, their respective numbers were separated by a slash. For methyl protons, the last number identifying each proton was dropped from the name. Geminal proton names were assigned to follow the ordering observed in the Biological Magnetic Resonance Data Bank (BMRB) statistics (https://bmrb.io/histogram/, last access: 1 April 2024) and do not reflect a stereospecific analysis of the fitted ${}^{3}J$ coupling values.

Fitting was done with the refit_peaks.R script from FitNMR 0.7. The spectra were fit in a region of ±0.02 ppm from the starting peaks in each multiplet. The chemical shift was allowed to move up to 3.5 times the starting $R_{2}^{*}$ during fitting. $R_{2}^{*}$ was constrained to being 0.1 to 2 Hz , and the scalar couplings were constrained to be -20 to 20 Hz .

### 2.3 Karplus parameters for side-chain χ angles

When spanning a rotatable bond, ${}^{3}J$ scalar couplings provide information about the dihedral angle ( θ ) between the two coupled atoms through the well-known Karplus relationship [Bibr bib1.bibx34]:

1 $${}^{3}J(\theta )=C_{0}+C_{1}\mathrm{cos}\theta +C_{2}\mathrm{cos}2\theta .$$

An alternative formulation of the Karplus relationship dependent on cos⁡θ and ${\mathrm{cos}}^{2}\theta$ terms is often used. Here, we apply two enhanced forms of the Karplus equation, one that is known as the generalized Karplus equation [Bibr bib1.bibx25] and another which we will refer to as the self-consistent Karplus equation [Bibr bib1.bibx44]. In this work, both are applied to $H_{1}-C_{1}-C_{2}-H_{2}$ dihedral angles in protein side chains. The generalized Karplus equation [Bibr bib1.bibx25] is

2 $$\begin{array}{rl} {}^{3}J(\theta ) & =P_{1}{\mathrm{cos}}^{2}\theta +P_{2}\mathrm{cos}\theta +P_{3}\\ & +\sum \Delta \chi_{i}^{g}\left(P_{4}+P_{5}{\mathrm{cos}}^{2}(\xi_{i}\theta +P_{6}|\Delta \chi_{i}^{g}|)\right). \end{array}$$

The $\Delta \chi_{i}^{g}$ terms give the electronegativity difference between the four other substituent groups ( $S_{1}$ to $S_{4}$ ) bonded to the central $C_{1}-C_{2}$ atom pair. They are calculated using the difference in Huggins electronegativity [Bibr bib1.bibx32] between hydrogen and the α atom (bonded to $C_{1}$ or $C_{2}$ ) and β atoms (bonded to the α atom) in each substituent group:

3 $$\Delta \chi^{g}=\Delta \chi^{\alpha }-P_{7}\sum \Delta \chi_{j}^{\beta }.$$

Here, we follow the geometric standard described by [Bibr bib1.bibx25], where $S_{1}$ and $S_{2}$ are bonded to $C_{1}$ , with $S_{1}$ directly clockwise from $H_{1}$ on a Newman projection, with $C_{1}$ in front of $C_{2}$ , and $S_{2}$ directly counterclockwise from $H_{1}$ . $S_{3}$ and $S_{4}$ are directly clockwise and counterclockwise, respectively, from $H_{2}$ on a Newman projection, with $C_{2}$ in front of $C_{1}$ . $\xi_{i}$ gives the sign of rotation and is +1 for $S_{1}/S_{3}$ and -1 for $S_{2}/S_{4}$ .

Parameters $P_{1}$ to $P_{7}$ were derived from fits to couplings in primarily six-membered ring structures with restricted geometries [Bibr bib1.bibx25]. Parameter set B ( $P_{1}=13.7$ , $P_{2}=-0.73$ , $P_{3}=0$ , $P_{4}=0.56$ , $P_{5}=-2.47$ , $P_{6}=16.9$ °, and $P_{7}=0.14$ ) was derived from couplings with two to four substituents. Parameter set D ( $P_{1}=13.22$ , $P_{2}=-0.99$ , $P_{3}=0$ , $P_{4}=0.87$ , $P_{5}=-2.46$ , $P_{6}=19.9$ °, and $P_{7}=0$ ) was derived from couplings with three substituents. Parameter set E ( $P_{1}=13.24$ , $P_{2}=-0.91$ , $P_{3}=0$ , $P_{4}=0.53$ , $P_{5}=-2.41$ , $P_{6}=15.5$ °, and $P_{7}=0.19$ ) was derived from couplings with four substituents. Here, we follow recommendations by [Bibr bib1.bibx25] using parameter sets B, D, and E for couplings with two, three, and four substituents, respectively.

For this work, we determined the complete set of parameters ( $\Delta \chi_{1}^{g}$ to $\Delta \chi_{4}^{g}$ ) necessary for a generalized Karplus analysis of proton–proton couplings associated with $\chi_{1\text{–}4}$ by analyzing representative amino acid structures taken from the PDB Chemical Component Directory (CCD) [Bibr bib1.bibx59]. An example representative structure for isoleucine is shown in Fig. [Fig Ch1.F1]a, and the parameters determined for all amino acids are given in Table [Table App1.Ch1.S1.T5] in Appendix A.

The self-consistent Karplus equation [Bibr bib1.bibx46] perturbs the average scalar coupling given by the $C_{0}$ coefficient in Eq. ([Disp-formula Ch1.E1]) using a set of increments ( $\Delta C_{0,i}$ ) weighted by the number ( $N_{i}$ ) of proton/heavy-atom α substitutions made around the bond for a particular element type i :

4 $${}^{3}J(\theta )=C_{0}+\sum (N_{i}\Delta C_{0,i})+C_{1}\mathrm{cos}\theta +C_{2}\mathrm{cos}2\theta .$$

This formulation makes it possible to extrapolate the parameterization to chemical substructures outside the training set. For side-chain proton–proton ${}^{3}J$ couplings, the following previously determined [Bibr bib1.bibx44] coefficients and coefficient increments were used: $C_{0}=7.24$ , $C_{1}=-1.37$ , $C_{2}=3.61$ , $\Delta C_{0,C}=0.61$ , $\Delta C_{0,O}=-1.59$ , and $\Delta C_{0,S}=-1.30$ Hz . The offset for nitrogen atoms was previously defined as $\Delta C_{0,N}=0$ Hz because $N_{N}=1$ for all side-chain $\chi_{1}$ angles, making it impossible to separate the contribution of a nitrogen substitution from the fundamental Karplus coefficient, $C_{0}$ . For the $\chi_{1}$ dihedral angle, the heavy-atom substitution counts were previously published [Bibr bib1.bibx44]. Here, we determined the number of α substituents for proton–proton couplings associated with $\chi_{1\text{–}4}$ , which are given in given in Table [Table App1.Ch1.S1.T6].

In Eqs. ([Disp-formula Ch1.E1]), ([Disp-formula Ch1.E2]), and ([Disp-formula Ch1.E4]), the θ angle refers to the dihedral angle between the two coupled protons, which is often offset from the canonical side-chain χ angle ( χ ) by a given value, Δχ :

θ=χ+Δχ.

In this paper, whenever the χ symbol has a superscript (like g, α , or β ), it refers to electronegativity. All other instances of χ refer to side-chain dihedral angles. Using the CCD representative amino acid structures, we determined Δχ offsets (rounded to -120 , 0, and 120°) for χ angles 1–4, which are given in given in Tables [Table App1.Ch1.S1.T5] and [Table App1.Ch1.S1.T6].

A comparison of the generalized and self-consistent Karplus equations is shown in Fig. [Fig App1.Ch1.S1.F7] in Appendix A. Over the nine sets of unique self-consistent parameters, the range of coupling values sampled by the generalized parameters is 2.7 Hz greater on average than the self-consistent parameters. Although the generalized Karplus equation was parameterized on bonds geometrically restricted by rings, the self-consistent equation was parameterized using data from a protein in solution. While efforts were made to account for the effects of protein motional averaging in the self-consistent parameterization (see below), it could be that the degree of protein motion was underestimated, resulting in less extreme Karplus curves in order to reproduce the measured scalar couplings. The generalized parameters also produce slightly higher couplings overall, with an average coupling value 0.6 Hz greater than the self-consistent parameters. Finally, averaging over the nine sets, there is a 1.5 Hz root mean square deviation between the couplings produced by the two equations.

### 2.4 χ angle distribution analysis

During self-consistent parameterization of the side-chain Karplus parameters [Bibr bib1.bibx44], two different models of motion were previously used. Model $M_{1}$ involved normally distributed fluctuation about a mean $\chi_{1}$ angle with standard deviation $\sigma_{\chi_{1}}$ . Model $M_{2}$ assumed jumps between 60, 180, and 300° and varied the respective populations. Each model had two free parameters, and $M_{1}$ was used for determining the final published parameters.

Here, we also apply a third model (which we call $M_{3}$ ) involving jumps between three rotameric bins, whose χ angle distributions were taken from the 2010 Dunbrack rotamer library [Bibr bib1.bibx47]. The $M_{3}$ model used 610 177 different side-chain conformations from their dataset. For each side-chain conformation, the theoretical coupling value was calculated using Eq. ([Disp-formula Ch1.E4]) and the data from Table [Table App1.Ch1.S1.T6]. Depending on the application, these theoretical couplings were averaged over all rotamers (as done for Fig. [Fig Ch1.F3]) or the three different rotameric bins associated with $\chi_{1}$ (as done for Fig. [Fig Ch1.F5]).

## 3 Results and discussion

### 3.1 Fitting amino acid one-dimensional spectra

To gain an insight into coupling patterns between carbon-bound protons in amino acid side chains, we performed fits of spectra taken from the Biological Magnetic Resonance Data Bank (BMRB) [Bibr bib1.bibx29] (Table [Table App1.Ch1.S1.T4]). The samples contained individual amino acids dissolved in $D_{2}O$ , nearly eliminating peaks from solvent and exchangeable protons. A representative fit for isoleucine is shown in Fig. [Fig Ch1.F1]. The resonances are defined as shown in Table [Table Ch1.T1] and parameters derived from the fit are shown in Tables [Table Ch1.T1]–[Table Ch1.T3]. The HA, HG2, and HD1 resonances are each affected by only one or two ${}^{3}J$ couplings, making their relatively simple multiplet patterns easy to resolve. The HG12 and HG13 resonances add a mutual ${}^{2}J$ coupling and a ${}^{3}J$ coupling to the HB atom. The HG12/HG13 chemical shift difference of 104.7 Hz (relative to the -13.5 Hz ${}^{2}J$ coupling) is sufficient to minimize roofing effects from strong coupling in the experimental data (black), which shows minimal deviation from the modeled contribution of each resonance (gray and yellow, respectively). Despite a very complicated multiplet pattern for the HB atom (a doublet of doublets of doublets of quartets; blue), the resonance is very well fit by the model due to the couplings being shared with resonances having much less complexity.

**Figure 1 Ch1.F1:**
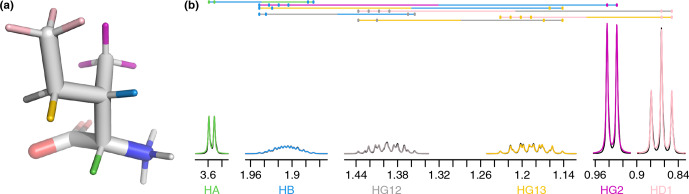
**(a)** Representative structure of isoleucine taken from the CCD with the termini made zwitterionic in PyMOL. Protons are grouped by color, with each color having a distinct chemical shift modeled by a single resonance in the fit. The ${\mathrm{NH}}_{3}^{+}$ hydrogens (white) are deuterated due to exchange with $D_{2}O$ . **(b)** Fit of 500 MHz ${}^{1}H$ NMR spectrum of isoleucine in $D_{2}O$ as described by Tables [Table Ch1.T1]–[Table Ch1.T3]. The experimental spectrum is shown in black, and the modeled signal corresponding to each resonance is shown with the same color as in **(a)**. Each scalar coupling is represented by a horizontal line above the spectrum, with the colors matched to the group being coupled to. The outermost multiplet produced by each coupling is represented by vertical dashes. The x axis gives the ${}^{1}H$ chemical shift in parts per million using sparse representation. This panel was produced with the plot_sparse_1d FitNMR function.

**Figure 2 Ch1.F2:**
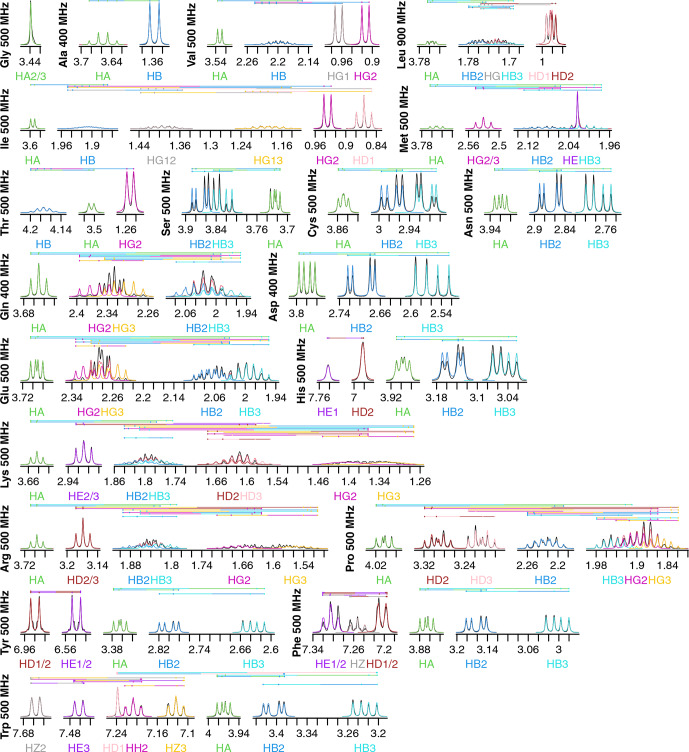
Fits of ${}^{1}H$ NMR spectra of all 20 canonical amino acids in $D_{2}O$ . Spectra are plotted as described in Fig. [Fig Ch1.F1]. The total modeled sum of all resonance contributions is shown in red, which is usually obscured by the individual contributions.

Fits for all 20 amino acids are shown in Fig. [Fig Ch1.F2]. Similar to isoleucine, the relatively simple spectra for glycine, alanine, valine, and threonine are all fit quite well and do not show significant strong coupling effects. The same is true of tryptophan, which has eight distinct resonances but only very slight strong coupling between HB2 and HB3.

Strong coupling is more pronounced in the beta protons of serine, cysteine, asparagine, and aspartate. These four side chains have the same three protons in the spectra, with HB2 and HB3 showing significant roofing effects. However, the multiplet patterns are easily resolved, and the weak-coupling model used by FitNMR finds an intermediate intensity between the two doublets. Despite not modeling the roofing effect, the line widths do not appear to be distorted by the intensity mismatch. Histidine is largely similar with the addition of HD2 and HE1 nuclei in the imidazole ring that are only coupled to one another via a 1.7 Hz ${}^{4}J$ coupling.

Glutamine and glutamate add HG2 and HG3 nuclei, each with similar but distinct chemical shifts, leading to large strong coupling effects. This results in outer multiplet peaks nearly disappearing. In proline, the HG2 and HG3 nuclei also have very similar chemical shifts and are quite strongly coupled. For methionine, the HG2 and HG3 nuclei appear to have indistinguishable chemical shifts and very similar scalar couplings, producing two overlapping, near-canonical triplets.

Leucine, with one HG atom, has two terminal methyl groups, each represented by a single resonance (HD1 or HD2). These make the multiplet pattern for HG quite complex. Due to the very similar ${}^{3}J$ (HG–HD1) and ${}^{3}J$ (HG–HD2) coupling constants (6.6 and 6.5 Hz, respectively), it is essentially a doublet of doublets of septets. Together with significant overlap between HB2, HB3, and HG (forming a strong coupling network between the three nuclei), this makes fitting the spectrum in this region very difficult. However, it is made somewhat easier because couplings involving the more isolated HA, HD1, and HD2 can be more easily resolved.

Tyrosine and phenylalanine also have somewhat complicated coupling networks in their aromatic rings, with four and five strongly coupled nuclei, respectively. They should each theoretically have both ${}^{3}J$ (HD1–HE1 or HD2–HE2) and ${}^{5}J$ (HD1–HE2 or HD2–HE1) couplings. In a purely weak-coupling model neglecting couplings between equivalent nuclei, that would create a doublet of doublets for HD1/2 and HE1/2 in tyrosine. However, the experimental spectrum (black) resembles a doublet of triplets. The outer peaks in each triplet have much lower intensities than a classic 1 : 2 : 1 triplet and exhibit roofing. Accurate modeling of this requires separate quantum mechanical treatment of the spin states of HD1, HD2, HE1, and HE2. During fitting, ${}^{5}J$ (HD–HE) drops to less than 0.0001 Hz, represented by the topmost vertical line. The phenylalanine fit does obtain reasonable values of 1.2 and 0.7 Hz for ${}^{5}J$ (HD–HE) and ${}^{4}J$ (HD–HZ), respectively. However, triplet behavior is still observed in the experimental spectrum, particularly for HE1/2, and remains unexplained by the weak-coupling model.

Lysine, arginine, and proline are all capable of having distinct proton chemicals shifts at the beta, gamma, and delta positions. Distinct chemical shifts are observed for all such protons except for the HD2 and HD3 atoms in arginine. They show identical chemical shifts and produce a near-perfect triplet, suggesting that the scalar couplings they make with HG2 and HG3 rotationally average out to near-identical values. The values of those scalar couplings and rotational averaging will be discussed in more detail below. While nearly all resonances in proline are modeled well, lysine and arginine are more difficult, especially for the HG2 and HG3 atoms, each of which are coupled to five nuclei.

Our data show that the large majority of protons in amino acid side chains can be modeled well using the FitNMR weak-coupling approximation. However, peak overlap is an issue for several nuclei, suggesting two-dimensional proton spectra like a nuclear Overhauser effect spectroscopy (NOESY) or double-quantum-filtered COSY (DQF-COSY) may be required for adequate resolution. In addition, FitNMR and similar methods would benefit from the incorporation of quantum mechanical calculations to enable accounting for strong coupling in the spectra.

### 3.2 χ -angle-dependent side-chain scalar couplings

Karplus parameters are required to derive structural information from scalar couplings. For ${}^{3}J$ couplings between adjacent ${\mathrm{CH}}_{2}$ groups, the four proton–proton couplings completely sample all three values of Δχ (see $\chi_{2\text{–}4}$ parameters in Tables [Table App1.Ch1.S1.T5] and [Table App1.Ch1.S1.T6]), providing detailed structural information. To use the generalized Karplus equation, we calculated the required electronegativity differences and positions of all substituent groups (Table [Table App1.Ch1.S1.T5]). For the self-consistent Karplus equation, we extrapolated parameters derived from scalar couplings associated with $\chi_{1}$ [Bibr bib1.bibx44] to $\chi_{2\text{–}4}$ (Table [Table App1.Ch1.S1.T6]). We did not attempt a reparameterization of $C_{0}$ and $\Delta C_{0,N}$ (see Methods, Sect. [Sec Ch1.S2.SS3]) to account for the absence of a nitrogen substitution at $\chi_{2}$ (in leucine, isoleucine, methionine, glutamine, glutamate, lysine, arginine, and proline) or $\chi_{3}$ (in lysine).

**Figure 3 Ch1.F3:**
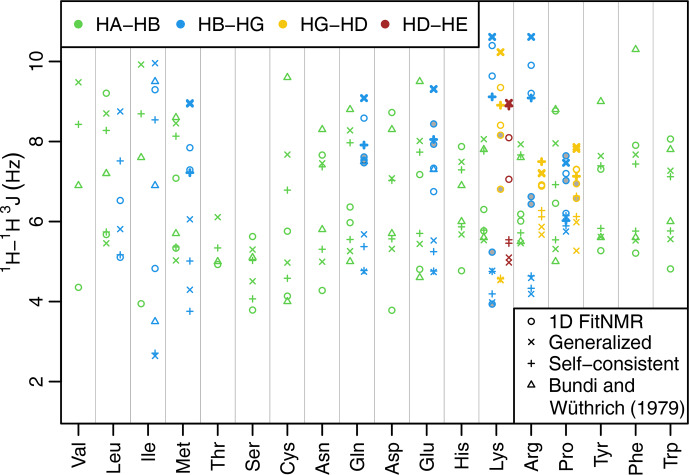
Side-chain ${}^{1}H{-}^{1}H$ ${}^{3}J$ scalar couplings that depend on χ angles through the Karplus relationship. Couplings from fits of the one-dimensional NMR spectra are shown with circles. Theoretical couplings calculated from the dataset used to create the 2010 Dunbrack rotamer library are shown with an × (using generalized Karplus equation) or a plus sign (using self-consistent equation). Experimental couplings from GGXA tetrapeptides ([Bibr bib1.bibx4]) are shown with triangles. HA–HB couplings are shown in green, HB–HG couplings are shown in blue, HG–HD couplings are shown in yellow, and HD–HE couplings are shown in auburn. The dihedral angles governing ${}^{3}J$ (HB2–HG2) and ${}^{3}J$ (HB3–HG3) have the same Δχ (see Methods and Tables [Table App1.Ch1.S1.T5] and [Table App1.Ch1.S1.T6]) and therefore have the same theoretical value shown with a thick × or plus sign. The same is true for the corresponding HG–HD and HD–HE couplings. Experimental couplings between speculatively assigned atoms 2–2 and 3–3 of adjacent methylene groups are shaded gray. Depending on the actual assignments, either the shaded or the unshaded pair of experimental couplings should correspond to the theoretical couplings indicated with the thick symbols.

The fit-derived ${}^{3}J$ couplings dependent on a side-chain χ angle are shown as circles in Fig. [Fig Ch1.F3]. There are eight amino acids with $\chi_{2}$ -related couplings (blue), three amino acids with $\chi_{3}$ -related couplings (yellow), and one with $\chi_{4}$ -related couplings (auburn). For χ angles with ${\mathrm{CH}}_{2}$ groups on both sides of the associated rotatable bond, there are two couplings that in principle should take the same value due to having the same Δχ offset (see Methods, Tables [Table App1.Ch1.S1.T5] and [Table App1.Ch1.S1.T6], and Fig. [Fig App1.Ch1.S1.F7]h and i for possible exceptions). These scalar couplings were obtained without any constraint on their similarity in the software nor human knowledge of the expected equivalence during manual optimization of the input parameters. Despite that and the lack of stereospecific assignments, such equivalent couplings were within about 1 Hz of each other in all but one case (lysine HG–HD), supporting the relative accuracy of our approach despite the limitations.

As an initial point of comparison, we used the 2010 Dunbrack rotamer library dataset to calculate theoretical scalar couplings assuming the same χ angle distributions observed in crystal structures. In Fig. [Fig Ch1.F3], these are shown using either an × (calculated using generalized Eq. [Disp-formula Ch1.E2]) or a plus sign (calculated with self-consistent Eq. [Disp-formula Ch1.E4]). For the calculated rotamer library couplings, thick symbols represent the two scalar couplings with equivalent Δχ offsets. If our speculative stereospecific assignments for both methylene protons are either both correct or both incorrect, this pair of geometrically equivalent experimental coupling values are shown as shaded circles. Alternatively, if only one of the methylene assignments is incorrect, then the unshaded circles should be equivalent. For these geometrically equivalent couplings, the experimental and rotamer library couplings are generally within about 1.5 Hz of each other. As noted above, there are possible exceptions to the coupling equivalence that happen due to geometric relationships between electron-withdrawing groups and the coupled protons [Bibr bib1.bibx25], which is accounted for by the generalized Karplus equation. However, the only angle where this is observable in our simulated couplings is at proline $\chi_{3}$ (see the two distinct thick yellow × symbols for proline in Fig. [Fig Ch1.F3] and phase-shifted solid lines in Fig. [Fig App1.Ch1.S1.F7]i).

Beta-branched amino acids have just a single coupling associated with $\chi_{1}$ , allowing unambiguous comparison. Of these, the experimental and rotamer library couplings are very similar for threonine. However, the couplings for valine and isoleucine are quite different, suggesting that some combination of the charged termini, absence of neighboring amino acid residues, or solvent exposure alters the free energy of these hydrophobic residues when free in solution. For valine and isoleucine, GGXA tetrapeptide couplings [Bibr bib1.bibx4] are closer to the rotamer-library-derived couplings than the free amino acid couplings. For other residues, notably cysteine, glutamate, tyrosine, and phenylalanine, one of the tetrapeptide couplings is much higher than any of the free amino acid or rotamer couplings.

Many of the experimentally measured scalar couplings with ambiguous assignments have rotamer library values somewhat nearby, providing less support for (but not necessarily excluding) differences in the energetic preferences. One possible systematic divergence between the experimental and rotamer-derived couplings was in the absolute difference between the two HA–HB couplings, $\Delta {}^{3}J\text{(HA–HB)}=|{}^{3}J\text{(HA–HB2)}-{}^{3}J\text{(HA–HB3)}|$ , which is especially pronounced for aspartate. However, with the experimental $\Delta {}^{3}J\text{(HA–HB)}$ value being greater than the rotamer library value (calculated using self-consistent Eq. [Disp-formula Ch1.E4]) for 10 out of 15 residues, the difference was not statistically significant ( p=0.20 ). Likewise, using generalized Karplus Eq. ([Disp-formula Ch1.E2]) to calculate rotamer library values, only 8 out of 15 residues showed a larger experimental coupling range ( p=0.80 ).

### 3.3 Analysis of $\chi_{1}$ angle distributions

To more quantitatively model distributions of the $\chi_{1}$ angle, for which the most reliable Karplus parameters were available, we used several different models of motion. The first, $M_{1}$ , models χ angle fluctuations as being normally distributed with standard deviations ranging from 0–50°. During development of the self-consistent Karplus parameters, both the Karplus parameters and the $M_{1}$ model parameters ( $\chi_{1}$ and $\sigma_{\chi_{1}}$ ) describing each experimentally measured residue were jointly optimized to be self-consistent with one another [Bibr bib1.bibx44].

**Figure 4 Ch1.F4:**
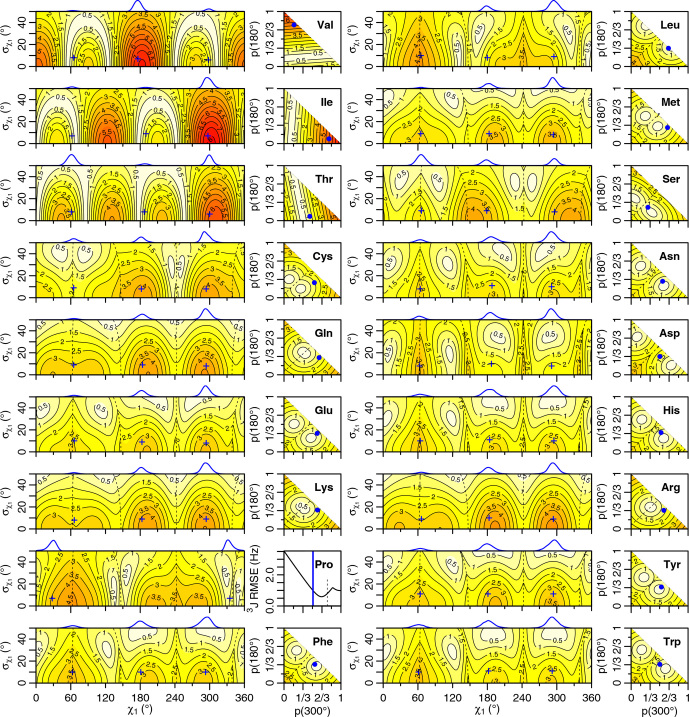
Generalized Eq. ([Disp-formula Ch1.E2]) ${}^{3}J$ (HA–HB) root mean squared error (RMSE) in hertz for $M_{1}$ (left) and $M_{3}$ (right) models of motion. Dashed lines separate regions with swapped assignments. Dunbrack rotamer library distributions are shown on top of the $M_{1}$ plots, with the $M_{1}$ model having the closest match to each shown with a blue plus sign. Rotamer library populations are shown as a blue point or solid vertical line in the $M_{3}$ plots.

**Figure 5 Ch1.F5:**
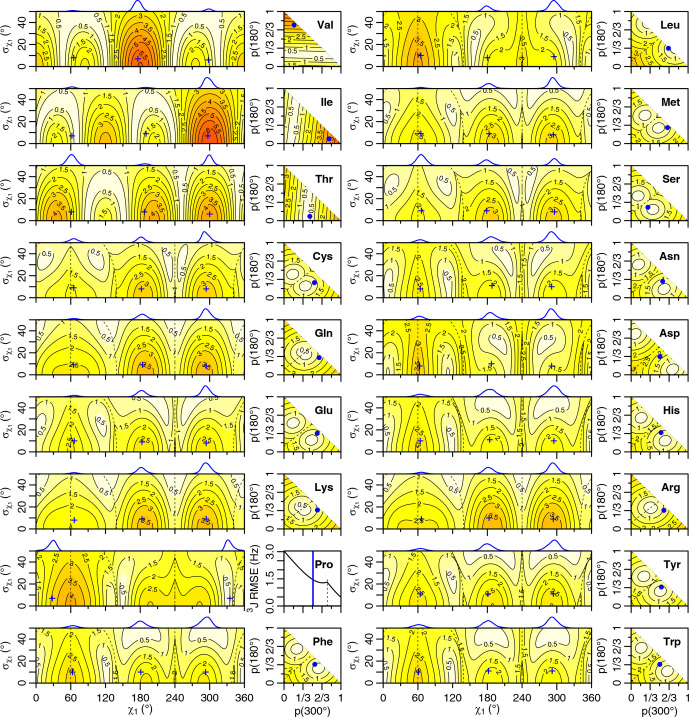
Self-consistent Eq. ([Disp-formula Ch1.E4]) ${}^{3}J$ (HA-HB) root mean squared error (RMSE) in hertz for $M_{1}$ (left) and $M_{3}$ (right) models of motion. Dashed lines separate regions with swapped assignments. Dunbrack rotamer library distributions are shown on top of the $M_{1}$ plots, with the $M_{1}$ model having the closest match to each shown with a blue plus sign. Rotamer library populations are shown as a blue point or solid vertical line in the $M_{3}$ plots.

For the full range of $\chi_{1}$ and $\sigma_{\chi_{1}}$ values, we calculated the root mean squared error (RMSE) between the back-calculated and experimentally measured scalar couplings. Those are shown as rectangular contour plots in Fig. [Fig Ch1.F4] (generalized Eq. [Disp-formula Ch1.E2]) and Fig. [Fig Ch1.F5] (self-consistent Eq. [Disp-formula Ch1.E4]). For the evaluation of the different models, we made no assumption about the stereospecific assignments of the HB2 and HB3 atoms. The RMSE values were calculated for both possible assignments, and the minimum RMSE for a given set of model parameters is shown. Boundaries between regions with different assignments are drawn as dashed lines. For the beta-branched amino acids (valine, isoleucine, and threonine), there is no such ambiguity, but the single scalar coupling provides less information.

The $\chi_{1}$ distributions used by the Dunbrack rotamer library are shown in blue on top of each contour plot. For reference, we determined the $M_{1}$ model parameters that produced the closest distribution (in terms of the Bhattacharyya distance) to each rotameric bin distribution. Those parameters are shown with blue plus signs. For amino acids excluding proline, the mean angles matching the rotamer library distributions (ranging from 61–66, 176–190, and 291–300°) were close to the canonical values. Due to the need for ring closure, the proline $\chi_{1}$ angle distributions are skewed towards 0 or 360° and report primarily on ring pucker. The standard deviations of the rotamer library distributions ranged 6–11°, with aromatic side chains having the most variation ( $\sigma_{\chi_{1}}\geq 10$ °).

While $\sigma_{\chi_{1}}$ was varied, 0–50°, both here and in the self-consistent Karplus parameterization, $\sigma_{\chi_{1}}$ values much greater than those observed in the PDB are not physically realistic. Furthermore, mean angles too far from those observed in the PDB are also not likely. The applicability of the unimodal $M_{1}$ model to the experimental data can be judged based on how nearby a region with low RMSE is to the blue plus sign. For nearly all of the amino acids, the measured ${}^{3}J$ (HA–HB) couplings are sufficient to exclude the $M_{1}$ model, suggesting that they instead populate multiple rotamer bins, as would be expected for an free amino acid in solution. Proline does show a set of $M_{1}$ parameters with low self-consistent RMSE values very close to a rotamer library distribution. Isoleucine is the only other amino acid where the single-rotamer model could be considered reasonable (with a plus symbol RMSE < 1 Hz), likely due to the reduced information content of the single scalar coupling.

An alternate $M_{2}$ model was previously tested that back-calculated the scalar couplings using a population-weighted mean of the theoretical scalar couplings at 60, 180, and 300°, which also makes it a two-parameter model [Bibr bib1.bibx44]. However, as the Dunbrack rotamer library indicates, side chains generally sample a range of values within a rotamer well. In addition, there is an amino-acid-specific bias away from the canonical angles, which can be subtle for many amino acids but quite large for proline. To account for this prior information, we propose another two-parameter model, referred to here as $M_{3}$ , that uses average scalar couplings calculated directly from the rotameric bins in the Dunbrack 2010 rotamer library dataset.

The RMSE values for the $M_{3}$ model are shown as square contour plots in Figs. [Fig Ch1.F4] and [Fig Ch1.F5], with the populations from the rotamer library shown as a blue point. Because only two valid rotamers exist for proline, the RMSE is plotted as a line against the population of the 300° (gauche minus) rotamer bin, with the rotamer library population shown as a vertical blue line.

For the $M_{1}$ model, which allows χ angles with unrealistically high potential energies, it is possible to judge model applicability by comparing it with rotamer library distributions (i.e., blue plus signs). However, because the $M_{3}$ model stays within observable χ angles by definition, there is not necessarily a means to assess model validity with a priori information. However, the vast majority of free amino acids do have scalar couplings reasonably consistent (RMSE < 1.5 Hz) with the rotamer library populations, which is not necessarily expected given the presence of the ${\mathrm{NH}}_{3}^{+}$ and ${\mathrm{COO}}^{-}$ groups, lack of neighboring amino acids, and high solvent exposure.

**Figure 6 Ch1.F6:**
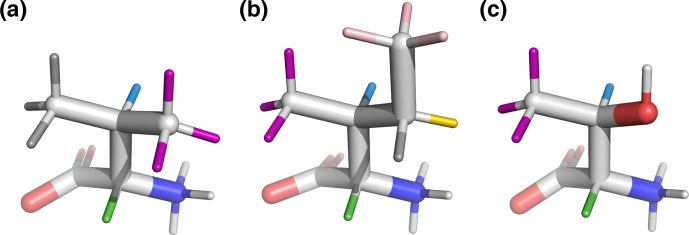
Beta-branched amino acids, with hydrogen colors matching those used in Figs. [Fig Ch1.F1] and [Fig Ch1.F2]. ${}^{3}J$ (HA–HB) is most sensitive to the population of the shown rotamers because HA (green) is trans to HB (blue), giving the maximum theoretical scalar coupling. **(a)** Valine, $\chi_{1}=180$ °, rotamer. **(b)** Isoleucine, $\chi_{1}=300$ °, rotamer. **(c)** Threonine, $\chi_{1}=300$ °, rotamer. Hydroxyl hydrogen (white) was also deuterated in the sample.

By contrast, beta-branched valine and isoleucine have ${}^{3}J$ (HA–HB) values (4.4 and 3.9 Hz , respectively) that are quite inconsistent with the rotamer populations observed in the PDB. The $\chi_{1}=180$ ° rotamer of valine and $\chi_{1}=300$ ° rotamer of isoleucine, both highly populated in the PDB, have very similar three-dimensional structures due to differences in the way $\chi_{1}$ atoms are defined (Fig. [Fig Ch1.F6]a, b). These rotamers are likely very prevalent in folded proteins because they avoid more strained conformations where either gamma carbon has two gauche interactions with the backbone. Interestingly, the threonine $\chi_{1}=300$ ° rotamer that has a similar heavy-atom arrangement (Fig. [Fig Ch1.F6]c) appears to have a 20 %–45 % population in solution according to the $M_{3}$ model (Figs. [Fig Ch1.F4] and [Fig Ch1.F5]). The differences in preferences for these rotamers in the free amino acids could arise due to the more hydrophobic side chains of valine and isoleucine imposing a greater desolvation penalty on the ${\mathrm{NH}}_{3}^{+}$ group than threonine does. The greater similarity of the GGXA tetrapeptide couplings to those from the rotamer library supports this mechanism (Fig. [Fig Ch1.F3]).

Proline is another amino acid whose PDB populations show varying levels of consistency with those observed for the free amino acid. Crystal structures show nearly equal populations of the $C_{\gamma }$ exo ( $\chi_{1}\approx 30$ °) and $C_{\gamma }$ endo ( $\chi_{1}\approx 330$ °) conformations. The interpretation of the solution NMR for free proline depends on the parameters used, with generalized Eq. ([Disp-formula Ch1.E2]) showing a 2 : 1 exo : endo ratio in rough agreement with the 1 : 1 ratio calculated by [Bibr bib1.bibx27], while self-consistent Eq. ([Disp-formula Ch1.E4]) shows a strong preference for the exo conformation. Aspartate also shows a stronger preference for either the $\chi_{1}=180$ ° or the $\chi_{1}=300$ ° rotamers when free in solution than it does in folded crystal structures.

Finally, several amino acids show near-uniform populations of their three different $\chi_{1}$ rotamers in solution, including lysine, arginine, and glutamine. All three side chains have longer aliphatic substructures, $({\mathrm{CH}}_{2}{)}_{2\text{–}4}$ , and positively charged or polar head groups, which may contribute to the relatively equal rotameric free energies.

## 4 Conclusions

Our results indicate that for most nuclei, the weak-coupling assumption yields useful information about side-chain dihedral angles. Only a small subset of nuclei show roofing effects from strong coupling, and for nearly all that do, it results from a geminal ${}^{2}J$ coupling that does not contain readily quantifiable structural information. For the aliphatic regions of longer side chains, where nuclei have both ${}^{2}J$ and ${}^{3}J$ couplings, strong coupling has a larger impact on multiplet analysis. To fully capture the complexity of multiplet patterns observed for such amino acid side chains, a strong coupling model is required. Even in multidimensional spectra that have insufficient resolution to accurately quantify scalar couplings through computational analysis, having an accurate model of the asymmetry is likely important for quantifying the volumes of severely overlapped peaks, for instance in a two-dimensional or three-dimensional NOESY. As such, the incorporation of a quantum mechanical spin system model into FitNMR is currently in progress.

Both the generalized [Bibr bib1.bibx25] and self-consistent [Bibr bib1.bibx44] Karplus equation parameterizations appear to produce reasonable agreement between experiment and theory when extrapolated to $\chi_{2\text{–}4}$ , which were not part of the original training data. By mapping out the full parameter space of motion models assuming the absence ( $M_{1}$ ) or presence ( $M_{3}$ ) of multiple rotamers, much can be learned about side-chain motion or lack thereof. As we illustrate here, differentiating between the models requires a minimum of two scalar couplings per bond. While helpful for maximum information content, stereospecific assignments do not appear to be strictly necessary to demonstrate the presence of multiple rotamers. While there are multiple purely heavy-atom scalar couplings associated with the $\chi_{1}$ angle, the same is not true for most $\chi_{2\text{–}4}$ angles. This illustrates the power of proton spectral analysis and provides motivation for further development in this area.

As shown here, peak overlap is already an issue for interpreting coupling constants from one-dimensional spectra of individual amino acids. Overlap becomes prohibitive in one-dimensional spectra of folded proteins but can likely be overcome to a large extent through the use of multidimensional ${}^{1}H{-}^{1}H$ two-dimensional spectra like the NOESY, which contain at least one isolated cross-peak for many nuclei. Without the presence of isotopically labeled heteronuclei requiring decoupling, the receiver can be left open during direct-dimension acquisition, allowing access to the complete free induction decay (FID). For small, single-digit kDa proteins, the multiplet patterns may be accessible to software like FitNMR in a similar manner to the ${}^{3}J$ (H-HA) doublet [Bibr bib1.bibx14]. A relatively new class of proteins that size is that of computationally designed miniprotein binders, which are able to target therapeutically relevant proteins [Bibr bib1.bibx5] and also are quite accessible to NMR characterization [Bibr bib1.bibx15]. Larger proteins may benefit from a strategy analogous to previously employed techniques [Bibr bib1.bibx42] of analyzing in-phase data together with anti-phase data from experiments like the DQF-COSY, where the observed signal intensity is proportional to the degree of anti-phase splitting by the coupling active in the cross-peak [Bibr bib1.bibx13]. Such spectra have historically been applied to the assignment and analysis of smaller unlabeled polypeptides [Bibr bib1.bibx60] but not fully exploited for their structural information content. This study lays the groundwork for comprehensive modeling and structural interpretation of multiplets in multidimensional protein spectra.

## Supplement

10.5194/mr-5-103-2024-supplement The supplement related to this article is available online at: https://doi.org/10.5194/mr-5-103-2024-supplement.

## Supplement

10.5194/mr-5-103-2024-supplement
10.5194/mr-5-103-2024-supplement The supplement related to this article is available online at: https://doi.org/10.5194/mr-5-103-2024-supplement.

## Figures and Tables

**Figure A1 App1.Ch1.S1.F7:**
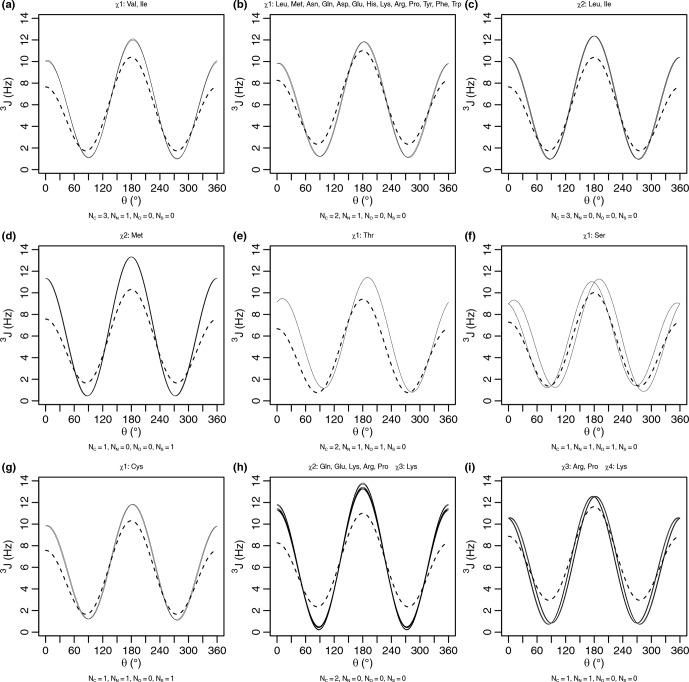
Generalized Karplus equation (thin lines) and self-consistent Karplus equation (dashed lines) for proton–proton ${}^{3}J$ scalar couplings across different χ angles. The number of atoms ( $N_{i}$ ) used for generating the self-consistent Karplus curves is given as a subtitle. The generalized Karplus curve phase shifts observed in **(f)** and **(i)** come from large differences in the electronegativity between substituents 3 and 4 (see Table [Table App1.Ch1.S1.T5]).

## Data Availability

This paper was prepared using R Markdown. All code and data required for reproducing the paper, figures, and tables in their entirety are available in the Supplement distributed with the paper. See the README.md file within for more details. The Supplement is also available at https://github.com/smith-group/syed2024 [Bibr bib1.bibx52], which may be updated as necessary to maintain software compatibility. The fitting methodology is implemented in the FitNMR open-source R package at https://github.com/smith-group/fitnmr [Bibr bib1.bibx49].

## Appendix A Additional information

### A1 Processing amino acid one-dimensional NMR data

Amino acid one-dimensional NMR free induction decay (FID) data were converted and processed using NMRPipe. FID conversion was performed using the bruker program, with chemical shift referencing done using the temperature dependence of the $H_{2}O$ chemical shift. Temperatures ranged from 298–306 K depending on the amino acid sample (see Table [Table App1.Ch1.S1.T4]). Spectra were processed with the following NMRPipe script, which includes a cosine window function and frequency domain polynomial baseline correction:
#!/bin/csh

nmrPipe -in test.fid \
| nmrPipe  -fn SP -off 0.5 -end 1.00  \
           -pow 1 -c 0.5              \
| nmrPipe  -fn ZF -auto               \
| nmrPipe  -fn FT -auto               \
| nmrPipe  -fn PS -p0 $P0 -p1 $P1     \
           -di -verb                  \
| nmrPipe  -fn POLY -auto             \
   -ov -out test.ft1

The zero- and first-order phases were extracted from the original TopSpin processing parameters. Their signs were changed prior to insertion into the NMRPipe script above. The Bruker PHC0 and PHC1 parameters were extracted using the following commands:

grep PHC0 pdata/1/proc | cut -d " " -f 2
grep PHC1 pdata/1/proc | cut -d " " -f 2

**Table A1 App1.Ch1.S1.T4:** Amino acid data used from the BMRB. Temperatures shown are from recorded Bruker acquisition parameters.

Amino	Field strength	Temperature	BMRB ID
acid	[MHz]	[K]	
Ala	400	306	bmse000028
Arg	500	298	bmse000029
Asn	500	298	bmse000030
Asp	400	306	bmse000031
Cys	500	298	bmse000034
Gln	400	306	bmse000038
Glu	500	298.16	bmse000037
Gly	500	298	bmse000089
His	500	298	bmse000039
Ile	500	298	bmse000041
Leu	900	298	bmse000042
Lys	500	298	bmse000043
Met	500	298	bmse000044
Phe	500	298	bmse000045
Pro	500	298	bmse000047
Ser	500	298	bmse000048
Thr	500	298	bmse000049
Trp	500	298	bmse000050
Tyr	500	298.16	bmse000051
Val	500	298	bmse000052

**Table A2 App1.Ch1.S1.T5:** Generalized Karplus equation parameters for ${}^{1}H$ - ${}^{1}H$ ${}^{3}J$ couplings. $\Delta \chi_{i}^{g}$ gives the difference in substituent group electronegativity.

AA	χ no.	Δχ	H ${}_{1}$	H ${}_{2}$	$\Delta \chi_{1}^{g}$	$\Delta \chi_{2}^{g}$	$\Delta \chi_{3}^{g}$	$\Delta \chi_{4}^{g}$	AA	χ no.	Δχ	H ${}_{1}$	H ${}_{2}$	$\Delta \chi_{1}^{g}$	$\Delta \chi_{2}^{g}$	$\Delta \chi_{3}^{g}$	$\Delta \chi_{4}^{g}$
Val	1	0	HA	HB	0.850	-0.094	0.400	0.400	Lys	2	0	HB2	HG2	0	0.225	0.344	0
Leu	1	-120	HA	HB2	0.850	0.400	0.400	0	Lys	2	120	HB2	HG3	0	0.225	0	0.344
Leu	1	0	HA	HB3	0.850	0.400	0	0.400	Lys	2	-120	HB3	HG2	0.225	0	0.344	0
Leu	2	120	HB2	HG	0	0.400	0.400	0.400	Lys	2	0	HB3	HG3	0.225	0	0	0.344
Leu	2	0	HB3	HG	0.400	0	0.400	0.400	Lys	3	0	HG2	HD2	0	0.344	0.281	0
Ile	1	-120	HA	HB	0.850	-0.094	0.324	0.400	Lys	3	120	HG2	HD3	0	0.344	0	0.281
Ile	2	0	HB	HG12	0.400	0.400	0.400	0	Lys	3	-120	HG3	HD2	0.344	0	0.281	0
Ile	2	120	HB	HG13	0.400	0.400	0	0.400	Lys	3	0	HG3	HD3	0.344	0	0	0.281
Met	1	-120	HA	HB2	0.850	0.400	0.400	0	Lys	4	0	HD2	HE2	0	0.344	0.850	0
Met	1	0	HA	HB3	0.850	0.400	0	0.400	Lys	4	120	HD2	HE3	0	0.344	0	0.850
Met	2	0	HB2	HG2	0	0.225	0.344	0	Lys	4	-120	HD3	HE2	0.344	0	0.850	0
Met	2	120	HB2	HG3	0	0.225	0	0.344	Lys	4	0	HD3	HE3	0.344	0	0	0.850
Met	2	-120	HB3	HG2	0.225	0	0.344	0	Arg	1	-120	HA	HB2	0.850	0.400	0.400	0
Met	2	0	HB3	HG3	0.225	0	0	0.344	Arg	1	0	HA	HB3	0.850	0.400	0	0.400
Thr	1	-120	HA	HB	0.850	-0.094	1.300	0.400	Arg	2	0	HB2	HG2	0	0.225	0.281	0
Ser	1	-120	HA	HB2	0.850	0.400	1.300	0	Arg	2	120	HB2	HG3	0	0.225	0	0.281
Ser	1	0	HA	HB3	0.850	0.400	0	1.300	Arg	2	-120	HB3	HG2	0.225	0	0.281	0
Cys	1	-120	HA	HB2	0.850	0.400	0.400	0	Arg	2	0	HB3	HG3	0.225	0	0	0.281
Cys	1	0	HA	HB3	0.850	0.400	0	0.400	Arg	3	0	HG2	HD2	0	0.344	0.794	0
Asn	1	-120	HA	HB2	0.850	0.400	0.400	0	Arg	3	120	HG2	HD3	0	0.344	0	0.794
Asn	1	0	HA	HB3	0.850	0.400	0	0.400	Arg	3	-120	HG3	HD2	0.344	0	0.794	0
Gln	1	-120	HA	HB2	0.850	0.400	0.400	0	Arg	3	0	HG3	HD3	0.344	0	0	0.794
Gln	1	0	HA	HB3	0.850	0.400	0	0.400	Pro	1	-120	HA	HB2	0.850	0.400	0.400	0
Gln	2	0	HB2	HG2	0	0.225	0.099	0	Pro	1	0	HA	HB3	0.850	0.400	0	0.400
Gln	2	120	HB2	HG3	0	0.225	0	0.099	Pro	2	0	HB2	HG2	0	0.225	0.281	0
Gln	2	-120	HB3	HG2	0.225	0	0.099	0	Pro	2	120	HB2	HG3	0	0.225	0	0.281
Gln	2	0	HB3	HG3	0.225	0	0	0.099	Pro	2	-120	HB3	HG2	0.225	0	0.281	0
Asp	1	-120	HA	HB2	0.850	0.400	0.400	0	Pro	2	0	HB3	HG3	0.225	0	0	0.281
Asp	1	0	HA	HB3	0.850	0.400	0	0.400	Pro	3	0	HG2	HD2	0	0.344	0.794	0
Glu	1	-120	HA	HB2	0.850	0.400	0.400	0	Pro	3	120	HG2	HD3	0	0.344	0	0.794
Glu	1	0	HA	HB3	0.850	0.400	0	0.400	Pro	3	-120	HG3	HD2	0.344	0	0.794	0
Glu	2	0	HB2	HG2	0	0.225	0.036	0	Pro	3	0	HG3	HD3	0.344	0	0	0.794
Glu	2	120	HB2	HG3	0	0.225	0	0.036	Tyr	1	-120	HA	HB2	0.850	0.400	0.400	0
Glu	2	-120	HB3	HG2	0.225	0	0.036	0	Tyr	1	0	HA	HB3	0.850	0.400	0	0.400
Glu	2	0	HB3	HG3	0.225	0	0	0.036	Phe	1	-120	HA	HB2	0.850	0.400	0.400	0
His	1	-120	HA	HB2	0.850	0.400	0.400	0	Phe	1	0	HA	HB3	0.850	0.400	0	0.400
His	1	0	HA	HB3	0.850	0.400	0	0.400	Trp	1	-120	HA	HB2	0.850	0.400	0.400	0
Lys	1	-120	HA	HB2	0.850	0.400	0.400	0	Trp	1	0	HA	HB3	0.850	0.400	0	0.400
Lys	1	0	HA	HB3	0.850	0.400	0	0.400									

**Table A3 App1.Ch1.S1.T6:** Self-consistent Karplus equation parameters for ${}^{1}H$ – ${}^{1}H$ ${}^{3}J$ couplings. $N_{i}$ gives the number of each type of substituent heavy atom.

AA	χ no.	Δχ	H ${}_{1}$	H ${}_{2}$	$N_{C}$	$N_{N}$	$N_{O}$	$N_{S}$	AA	χ no.	Δχ	H ${}_{1}$	H ${}_{2}$	$N_{C}$	$N_{N}$	$N_{O}$	$N_{S}$
Val	1	0	HA	HB	3	1	0	0	Lys	2	0	HB2	HG2	2	0	0	0
Leu	1	-120	HA	HB2	2	1	0	0	Lys	2	120	HB2	HG3	2	0	0	0
Leu	1	0	HA	HB3	2	1	0	0	Lys	2	-120	HB3	HG2	2	0	0	0
Leu	2	120	HB2	HG	3	0	0	0	Lys	2	0	HB3	HG3	2	0	0	0
Leu	2	0	HB3	HG	3	0	0	0	Lys	3	0	HG2	HD2	2	0	0	0
Ile	1	-120	HA	HB	3	1	0	0	Lys	3	120	HG2	HD3	2	0	0	0
Ile	2	0	HB	HG12	3	0	0	0	Lys	3	-120	HG3	HD2	2	0	0	0
Ile	2	120	HB	HG13	3	0	0	0	Lys	3	0	HG3	HD3	2	0	0	0
Met	1	-120	HA	HB2	2	1	0	0	Lys	4	0	HD2	HE2	1	1	0	0
Met	1	0	HA	HB3	2	1	0	0	Lys	4	120	HD2	HE3	1	1	0	0
Met	2	0	HB2	HG2	1	0	0	1	Lys	4	-120	HD3	HE2	1	1	0	0
Met	2	120	HB2	HG3	1	0	0	1	Lys	4	0	HD3	HE3	1	1	0	0
Met	2	-120	HB3	HG2	1	0	0	1	Arg	1	-120	HA	HB2	2	1	0	0
Met	2	0	HB3	HG3	1	0	0	1	Arg	1	0	HA	HB3	2	1	0	0
Thr	1	-120	HA	HB	2	1	1	0	Arg	2	0	HB2	HG2	2	0	0	0
Ser	1	-120	HA	HB2	1	1	1	0	Arg	2	120	HB2	HG3	2	0	0	0
Ser	1	0	HA	HB3	1	1	1	0	Arg	2	-120	HB3	HG2	2	0	0	0
Cys	1	-120	HA	HB2	1	1	0	1	Arg	2	0	HB3	HG3	2	0	0	0
Cys	1	0	HA	HB3	1	1	0	1	Arg	3	0	HG2	HD2	1	1	0	0
Asn	1	-120	HA	HB2	2	1	0	0	Arg	3	120	HG2	HD3	1	1	0	0
Asn	1	0	HA	HB3	2	1	0	0	Arg	3	-120	HG3	HD2	1	1	0	0
Gln	1	-120	HA	HB2	2	1	0	0	Arg	3	0	HG3	HD3	1	1	0	0
Gln	1	0	HA	HB3	2	1	0	0	Pro	1	-120	HA	HB2	2	1	0	0
Gln	2	0	HB2	HG2	2	0	0	0	Pro	1	0	HA	HB3	2	1	0	0
Gln	2	120	HB2	HG3	2	0	0	0	Pro	2	0	HB2	HG2	2	0	0	0
Gln	2	-120	HB3	HG2	2	0	0	0	Pro	2	120	HB2	HG3	2	0	0	0
Gln	2	0	HB3	HG3	2	0	0	0	Pro	2	-120	HB3	HG2	2	0	0	0
Asp	1	-120	HA	HB2	2	1	0	0	Pro	2	0	HB3	HG3	2	0	0	0
Asp	1	0	HA	HB3	2	1	0	0	Pro	3	0	HG2	HD2	1	1	0	0
Glu	1	-120	HA	HB2	2	1	0	0	Pro	3	120	HG2	HD3	1	1	0	0
Glu	1	0	HA	HB3	2	1	0	0	Pro	3	-120	HG3	HD2	1	1	0	0
Glu	2	0	HB2	HG2	2	0	0	0	Pro	3	0	HG3	HD3	1	1	0	0
Glu	2	120	HB2	HG3	2	0	0	0	Tyr	1	-120	HA	HB2	2	1	0	0
Glu	2	-120	HB3	HG2	2	0	0	0	Tyr	1	0	HA	HB3	2	1	0	0
Glu	2	0	HB3	HG3	2	0	0	0	Phe	1	-120	HA	HB2	2	1	0	0
His	1	-120	HA	HB2	2	1	0	0	Phe	1	0	HA	HB3	2	1	0	0
His	1	0	HA	HB3	2	1	0	0	Trp	1	-120	HA	HB2	2	1	0	0
Lys	1	-120	HA	HB2	2	1	0	0	Trp	1	0	HA	HB3	2	1	0	0
Lys	1	0	HA	HB3	2	1	0	0									

## Appendix

The supplement related to this article is available online at: https://doi.org/10.5194/mr-5-103-2024-supplement.
