# Supplementary material for: Analysis of chi angle distributions in free amino acids via multiplet fitting of proton scalar couplings
Source: Magn Reson (Gott). 2024 Aug 19;5(2):103–20. doi: 10.5194/mr-5-103-2024 (PMC11570886; doi:10.5194/mr-5-103-2024)
Supplement: The supplement related to this article is available online at: https://doi.org/10.5194/mr-5-103-2024-supplement. [file mr-5-103-2024-supplement.zip › mr-5-103-2024-supplement-title-page.pdf]

Supplement of Magn. Reson., 5, 103–120, 2024
https://doi.org/10.5194/mr-5-103-2024-supplement
© Author(s) 2024. CC BY 4.0 License.

Supplement of

Analysis of chi angle distributions in free amino acids via multiplet fitting of proton scalar couplings

Nabiha R. Syed et al.

Correspondence to: Colin A. Smith (colin.smith@wesleyan.edu)

- mr-5-103-2024-supplement-title-page.pdf
- Syed2024
  - DatasetForBBDepRL2010.txt
  - README.md
  - Syed2024.Rmd
  - Syed2024.bib
  - aa_images
    - beta.pml
    - ile.pml
  - copernicus.bst
  - copernicus.cfg
  - copernicus.cls
  - data
    - aa_images.tar.gz
    - aa_model.tar.gz
    - fit1d_fitnmr_output.tar.gz
    - fit1d_fitnmr_start.tar.gz
    - fit1d_gissmo.tar.gz
    - fit1d_nmrpipe_com.tar.gz
    - fit1d_nmrpipe_output.tar.gz
  - pdfscreen.sty
  - pdfscreencop.sty
  - scripts
    - aa_model_download.sh
    - fit1d_fitnmr_run.sh
    - fit1d_gissmo_download.sh

    * fit1d_gissmo_unzip.sh
    * fit1d_nmrpipe_run.sh
    * refit_peaks.R

The copyright of individual parts of the supplement might differ from the article licence.
